# S-ketamine as an adjuvant in patient-controlled intravenous analgesia for preventing postpartum depression: a randomized controlled trial

**DOI:** 10.1186/s12871-022-01588-7

**Published:** 2022-02-16

**Authors:** Yaqian Han, Pule Li, Mengrong Miao, Yuan Tao, Xia Kang, Jiaqiang Zhang

**Affiliations:** 1 Department of Anesthesiology and Perioperative Medicine, Henan University People’s Hospital; Henan Provincial People’s Hospital, Zhengzhou, China; 2 Department of Anesthesiology and Perioperative Medicine, Henan Provincial People’s Hospital, People’s Hospital of Zhengzhou University, Zhengzhou, 450003 China

**Keywords:** Cesarean section, Patient-controlled intravenous analgesia, PCIA, Postpartum depression, S-ketamine, Puerperal women

## Abstract

**Background:**

Postpartum depression (PPD) is a common complication of cesarean section. S-ketamine given intravenously during surgery can help prevent PPD. However, whether S-ketamine in patient-controlled intravenous analgesia (PCIA) can reduce the incidence of PPD is unknown. This study assessed the effect of S-ketamine as an adjuvant in PCIA for preventing PPD in women undergoing cesarean delivery.

**Methods:**

A total of 375 parturients scheduled to undergo cesarean section and then receive PCIA were recruited from a single center and were randomly assigned to control (C) group (sufentanil 2 μg/kg + tropisetron 10 mg) or S-ketamine (S) group (S-ketamine 0.5 mg/kg + sufentanil 2 μg/kg + tropisetron 10 mg). The primary outcome was the incidence of PPD measured by the Edinburgh postnatal depression scale (EPDS) after surgery. The secondary outcomes were EPDS scores, visual analog scale (VAS) scores, Ramsay sedation scale (RSS) scores, and the rate of adverse events, including headache, nausea, dizziness, drowsiness, and vomit.

**Results:**

A total of 275 puerperal women were included in the study. The rate of depression in parturient on postoperative days 3, 14, 28 in the C group and S group were 17.6 and 8.2% (*p* < 0.05), 24.2 and 9.8% (p < 0.05), and 19.0 and 17.2% (*p* = 0.76) respectively. EPDS scores in the C group and S group on postoperative days 3,14, and 28 were 7.65 ± 3.14 and 6.00 ± 2.47 (p < 0.05), 7.62 ± 3.14 and 6.38 ± 2.67 (p < 0.05), and 7.35 ± 3.17 and 6.90 ± 2.78 (*p* = 0.15), respectively. The rate of adverse events in the C group and S group were headache 3.3 and 4.1% (*p* = 0.755), nausea 5.9 and 8.2% (*p* = 0.481), dizziness 9.2 and 12.3% (*p* = 0.434), drowsiness 6.5 and 10.7%(*p* = 0.274), and vomit 5.9 and 5.7% (*p* = 0.585).

**Conclusions:**

S-ketamine (0.01 mg/kg/h) as an adjuvant in PCIA significantly reduces the incidence of PPD within 14 days and relieves pain within 48 h after cesarean delivery, without increasing the rate of adverse reactions.

**Trial registration:**

Registered in the Chinese Clinical Trial Registry (ChiCTR2100050263) on August 24, 2021.

## Background

Postpartum depression (PPD) is a common psychosocial disorder that can adversely affect the qualify of life of the mother, newborn, and family [1]. The reported incidence of PPD in China is 1.0–52.1% [2, 3]. Severe depression requiring hospitalization is more likely to occur after childbirth than at any other time in the lives of adult women [4] because childbirth is a powerful trigger for mania and psychosis. Furthermore, developing these conditions after childbirth can substantially increase the risk of suicide, which is a leading cause of maternal death [5]. Therefore, reducing the occurrence of PPD in puerperal women is crucial.

Ketamine effectively improves the symptoms of depression and reduces suicidal tendencies in depressed patients [1, 6, 7]. The intravenous injection of ketamine during cesarean delivery effectively prevents postpartum depression until 3 days to 1 month [1, 8]. As a dextral resolution of ketamine, the anesthetic effect of S-ketamine is two-fold higher than that of (R, S)-ketamine and approximately three-fold higher than that of (R)-ketamine [9–13]. Moreover, the rate of adverse events from S-ketamine was lower than that of subanesthetic doses of ketamine [14]. Therefore, we hypothesized that S-ketamine (0.01 mg/kg/h, infused continuously for 2 days) as an adjuvant in patient-controlled intravenous analgesia (PCIA) could prevent PPD.

## Methods

### Study design

The study was approved by the Research Ethics Committee of Henan Provincial People’s Hospital ([2019] 53), Henan, China, and was retroactive registered in the Chinese Clinical Trial Registry (ChiCTR2100050263) on August 24, 2021. All subjects gave written informed consent.

### Participants

Parturients admitted to Henan Provincial People’s Hospital were recruited from September 1, 2019, to July 15, 2020. The inclusion criteria were (1) women undergoing cesarean delivery in our hospital, (2) age of 18–45 years, (3) gestational age between 36 and 42 weeks, (4) BMI of 17–36 kg/m^2^, and (5) ASA grade I and II. The exclusion criteria were (1) prenatal depression (Edinburgh postnatal depression scale [EPDS] scores ≥10) diagnosed by a psychiatrist before enrollment, (2) pregnant women who experienced domestic violence, (3) serious obstetric complications, (4) women whose newborns had serious genetic or congenital diseases, and (5) multiparous women.

### Randomization and blinding

Parturients gave written informed consent on the day of cesarean delivery and were randomly assigned to a control group (C group) and an intervention group (S group) (1; 1) using SPSS version 25.0. Treatment allocation was performed by a nurse who gave PCIA. Anesthesiologists, researchers, and study participants were blinded to allocation. In this setting, blinded investigators reported adverse events, including headache, nausea, vomiting, dizziness, and drowsiness, to staff, who referred the patients to anesthesiologists for treatment based on symptoms.

### Procedures

All parturients underwent spinal anesthesia at L3-L4 or L2-L3 and received PCIA after surgery. The anesthetic solution (2 mL of 1% ropivacaine + 1 mL of 10% glucose) was administered by an anesthesiologist at a dose of 1 mL every 5 s to achieve the highest level of sensory block at T4–T6. At the end of the surgery, all patients received non-steroidal anti-inflammatory drugs (2.0 g of propacetamol intravenously) and preemptive analgesia of ultrasound-guided transversus abdominis plane block. PCIA included sufentanil 2 μg/kg + tropisetron 10 mg in the control group (C group) and sufentanil 2 μg/kg + tropisetron 10 mg + S-ketamine 0.5 mg/kg in the S-ketamine group (S group), in a total volume of 100 mL. All patients received continuous infusion (basal rate of 2 mL/h) and a 1-mL on-demand bolus with a lockout interval of 15 min. The infusion was started immediately after suturing the skin incision and lasted 48 h. The EPDS questionnaire was applied on the day of surgery in the preparation room to identify parturients with prenatal depression and on days 3, 14, and 28 after cesarean section to diagnose PPD. Moreover, on these days, parturients were contacted by phone or WeChat to complete EPDS as they were discharged from the hospital within 3 days after cesarean section. The EPDS contains ten questions, each worth up to 3 points, and the total score is 30. In our study, EPDS scores ≥10 were considered to indicate postpartum depression [3].

The VAS and RSS questionnaires were applied at 4, 8, 12, 24, and 48 h after cesarean delivery. Adverse events during PCIA, including headache, nausea, dizziness, drowsiness, and vomiting, were recorded.

### Outcome measures

The primary outcome was the incidence of PPD. The secondary outcomes were EPDS scores before surgery and at days 3, 14, and 28 after cesarean section. RSS and VAS scores were measured at 4, 8, 12, 24, and 48 h postpartum.

Adverse events during PCIA, including headache, nausea, dizziness, drowsiness, and vomiting, were recorded.

### Sample size and statistical analysis

The reported rate of postpartum depression (EPDS scores ≥10) in China is 28.2% [2, 3]. Assuming that ketamine reduces the rate of postpartum depression by 15%, the analysis using PASS version 15.0 with a statistical power of 80% and α = 0.05 for two-tailed tests yielded a sample size of 300 (150 participants in each group). Assuming a drop-out rate of 20%, 375 patients were expected to be recruited.

Continuous variables were compared using the independent-samples *t*-test or Mann-Whitney U test and were presented as the mean ± standard deviation or median (minimum and maximum). Categorical variables were compared using the chi-square test or Fisher’s exact test and were presented as percentages. Statistical analysis was performed using SPSS software version 25.0. A two-sided *p*-value of less than 0.05 was considered statistically significant.

## Results

**Fig. 1:**
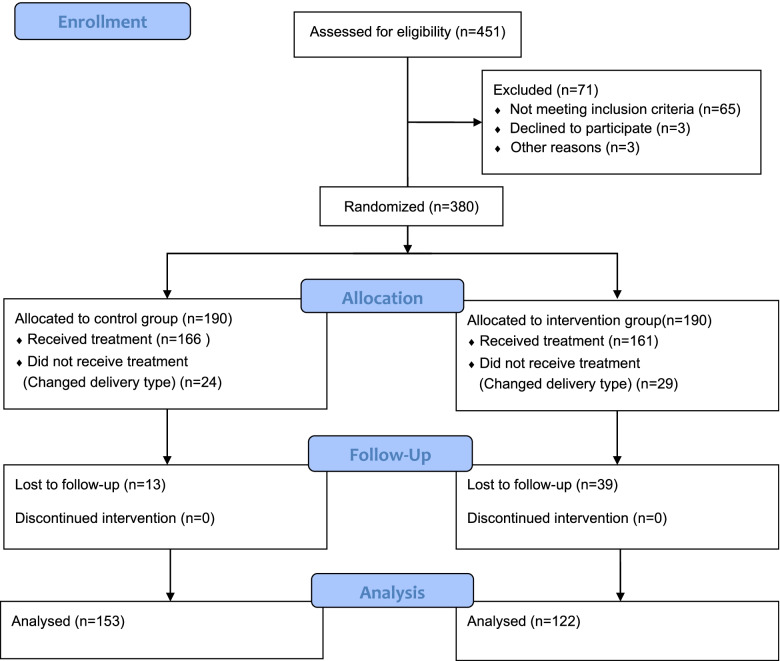
Flowchart of patient selection

A total of 451 parturients were recruited from September 1, 2019, to July 15, 2020. Of these, 71 underwent vaginal delivery, 53 refused to participate in the study after cesarean delivery, and 52 were lost to follow-up. Therefore, 275 patients were included in the analysis (Fig. 1).

### Baseline characteristics

The baseline characteristics of our cohort are shown in Table 1.

**Table 1:** Characteristics of our cohort

	C group(***n*** = 153)	S group(***n*** = 122)	***P***-value
Age, years	31.85 ± 4.16	31.64 ± 3.93	0.71
ASA physical status, I/II	39/114	31/91	1.0
BMI, kg/m^2^	26.89 ± 2.58	27.08 ± 2.95	0.63
Time of surgery, min	51.59 ± 11.07	53.60 ± 9.99	0.31
Blood loss, ml	301.83 ± 98.47	320.90 ± 121.26	0.73
EPDS score before surgery	6.54 ± 2.35	6.72 ± 2.25	0.48
Primiparous, yes/no	114/39	84/38	0.30
Education			0.19
Primary school	3	2	
Secondary school	26	10	
High school	48	41	
University	76	69	
Planned pregnancy, yes/no	137/16	108/14	0.34
Gender, boys/girls	81/72	77/45	0.66
Infant feeding^a^			0.12
Maternal	105	97	
Mixed	34	17	
Artificial	14	8	
Major family accident^a,b^	1	2	0.59

^a^ These data were obtained until day 28 after cesarean delivery, ^b^ Unemployment, death in the families, and other reasons

### Primary outcome

**Fig. 2:**
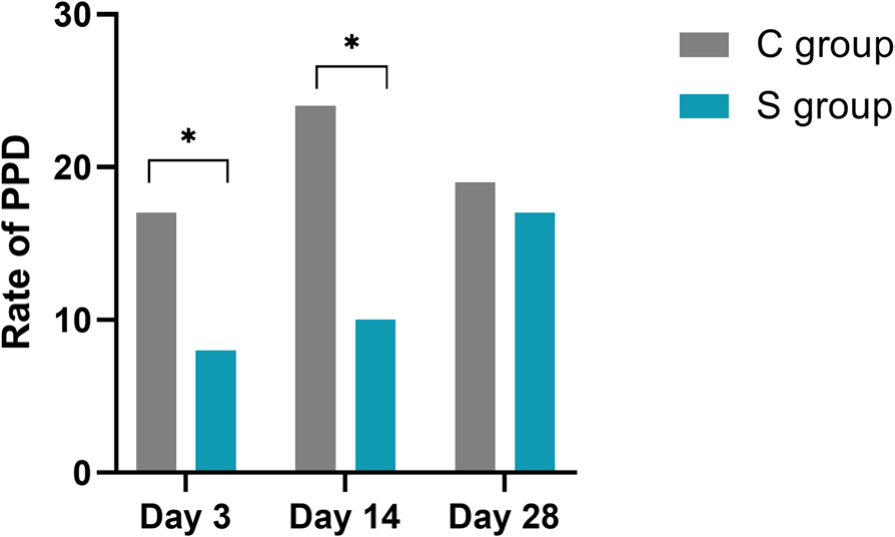
Rate of postpartum depression (PPD) after cesarean delivery. *P < 0.05

The rate of depression was significantly lower in the S group at 3 days and 14 days after cesarean section (17.6 and 8.2% vs. 24.2 and 9.8%, *p* < 0.05) (Fig. 2).

### Secondary outcome

**Fig. 3:**
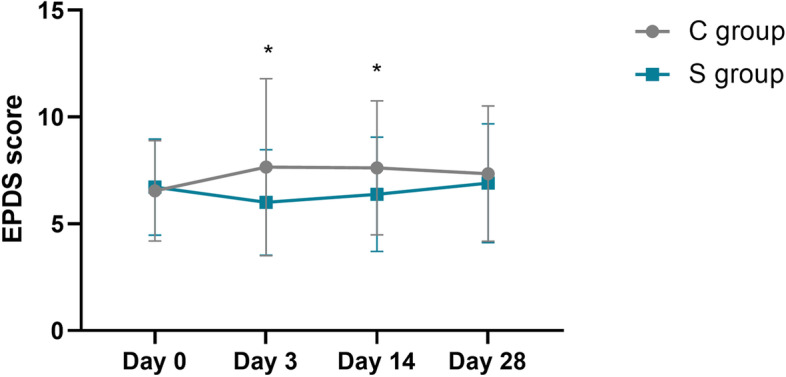
Edinburgh postnatal depression scale (EPDS) scores at different time points. *P < 0.05

EPDS scores were similar between the S group and C group before delivery (6.54 ± 2.35 vs. 6.72 ± 2.25, *P* = 0.508). However, there were significant intergroup differences in EPDS scores at 3 days (7.65 ± 3.14 vs. 6.00 ± 2.47, *p* < 0.001) and 14 days (7.62 ± 3.14 vs. 6.38 ± 2.67, p < 0.001) postpartum (Fig. 3).

**Fig. 4:**
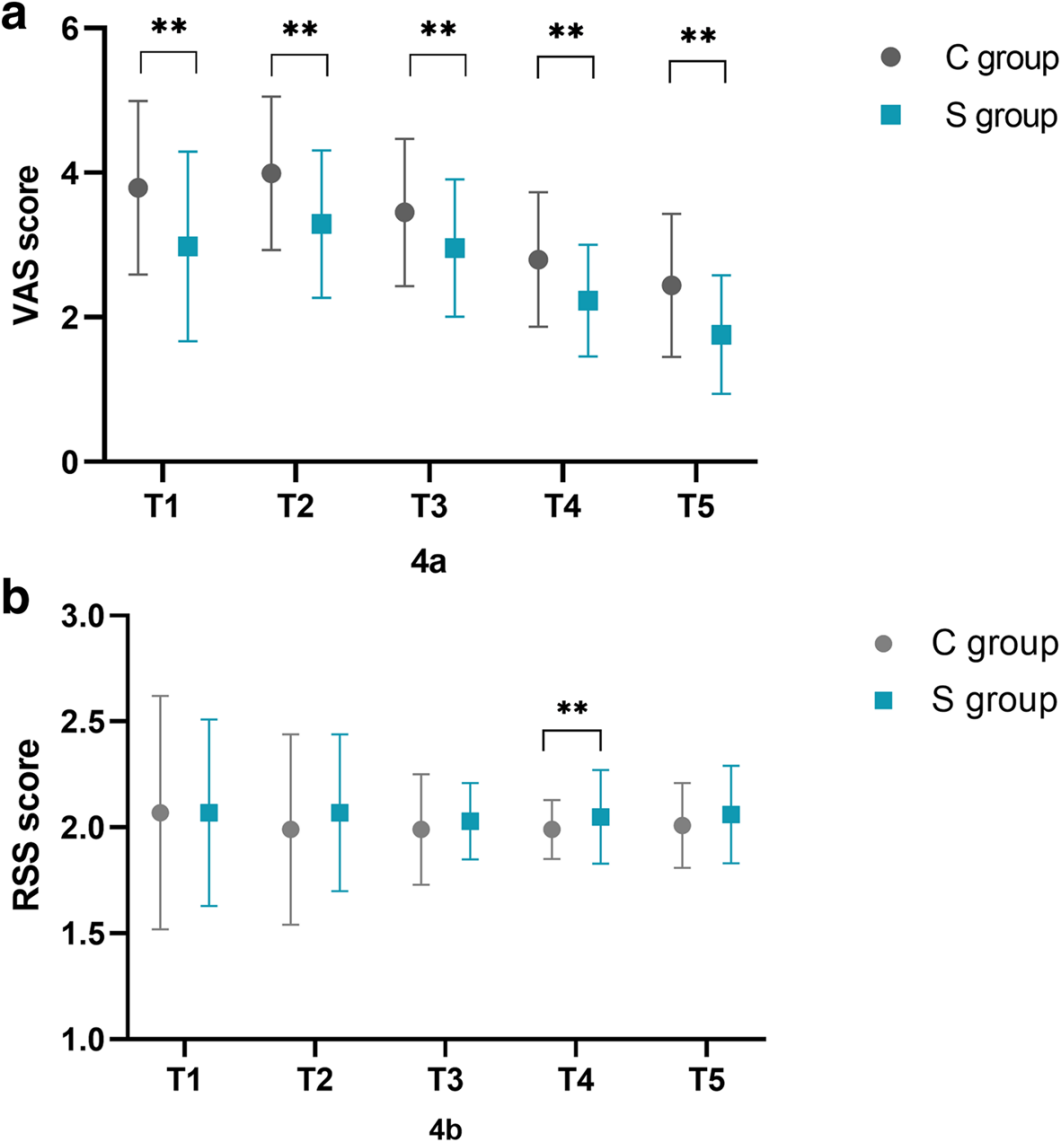
Visual analog scale (4a) and Ramsay sedation scale (4b) scores at different time points after cesarean delivery. **P < 0.05 at 4, 8, 12, 24, and 48 h (T1, T2, T3, T4) after the onset of patient-controlled intravenous analgesia

VAS scores were significantly lower in the S group at 4, 8, 12, and 24 h after cesarean section (Fig. 4a). RSS scores were similar between the groups, except at 24 h postpartum (1.99 ± 0.14 vs. 2.05 ± 0.22, *p* = 0.015) (Fig. 4b). The rate of adverse events was similar between the two groups (Table 2).

**Table 2:** Adverse events from patient-controlled intravenous analgesia

Adverse events	C group (%)	S group (%)	Total (%)	*P*-value
Headache	5 (3.3)	5 (4.1)	10 (3.6)	0.755
Nausea	9 (5.9)	10 (8.2)	19 (6.9)	0.481
Dizzy	14 (9.2)	15 (12.3)	29 (10.5)	0.434
Drowsiness	10 (6.5)	13 (10.7)	23 (8.4)	0.274
Vomit	9 (5.9)	7 (5.7)	16 (5.8)	0.585

## Discussion

The results showed that PCIA with S-ketamine (0.01 mg/kg/h) in women undergoing cesarean section reduces the incidence of PPD and EPDS scores within 14 days without increasing the rate of adverse events. In addition, PPD increased over time within 1 month in both groups, consistent with previous studies [1, 15, 16].

Ketamine rapidly and effectively reduces depressive symptoms postpartum [12, 17, 18]. PPD was effectively reduced at 1 week postpartum, but not at 2 and 4 weeks, in healthy women scheduled for cesarean delivery who received intravenous ketamine (0.25 mg/kg) intraoperatively. Furthermore, the rate of adverse events, including headache, hallucinations, and dizziness, was higher in the ketamine group during the operation. These data contradict our results to some extent, which may be due to differences in treatment protocols (single subanesthetic dose vs. continuous infusion), compliance adherence, demographic characteristics, and exclusion criteria.

Postpartum depression is usually defined as EPDS scores ≥10 [19, 20] and should be diagnosed within 6 weeks postpartum [21–25]. We used the Chinese version of the EPDS questionnaire, which has been validated for Chinese women. The administration of ketamine (0.5 mg/kg) 10 min after childbirth and 160 mg of ketamine in PCIA postoperatively effectively reduced PPD and postpartum blues within 4 days after cesarean delivery [16]. In line with this result, the rate of PPD in the S group decreased to some extent at 3 and 14 days postoperatively.

A low dose of ketamine reduces anesthetic side effects [14]. Ketamine used intraoperatively as an analgesic or sedative improved mood and depression scores in the postoperative period [7], consistent with our data. In our cohort, the VAS score during PCIA was significantly lower in the S group. In addition, the number of adverse events decreased to a certain extent in the S group.

A single intravenous dose of S-ketamine or ketamine during cesarean delivery may not affect the breastfed infant or lactation [3]. Based on the dose of S-ketamine administrated to our patients, we believe that the intravenous use of S-ketamine (approximately 0.01 mg/Kg/h) is safe for both mother and infant [26, 27].

This study has limitations. First, the sample size was small, and the study only included Chinese adults from a single center. Thus, larger multicenter and multi-ethnic cohort studies are warranted. Second, an S-ketamine dose of 0.5 mg/Kg was used in PCIA in our study, which is lower than the subanesthetic dose for treatment of depression [26–28]; thus, whether the effect of higher/lower doses could be better as for the safety and effectiveness in preventing and treating PPD should be further assessed. Third, PPD usually began within 1 month after delivery [29], hence, our patients were followed up for 28 days to evaluate the impact of S-ketamine on it. The long-term effects of the current intervention remain to be determined.

## Conclusions

The intravenous administration of S-ketamine (0.01 mg/kg/h) as an adjuvant in PCIA significantly reduces the incidence of PPD within 14 days after cesarean delivery and relieves pain within 48 h after delivery, without increasing the rate of adverse events, demonstrating the safety and effectiveness of this anesthetic in PCIA to improve PPD.

## Data Availability

The data supporting the findings of this study are not publicly available because of institutional policy but are available from the corresponding author on reasonable request.

## Abbreviations

ASA: American Society of Anesthesiology
EPDS: Edinburgh postnatal depression scale
PCIA: patient-controlled intravenous analgesia
PPD: Postpartum depression
RSS: Ramsay sedation scale
VAS: visual analog scale
